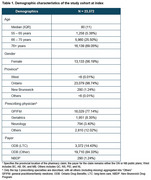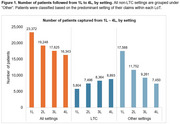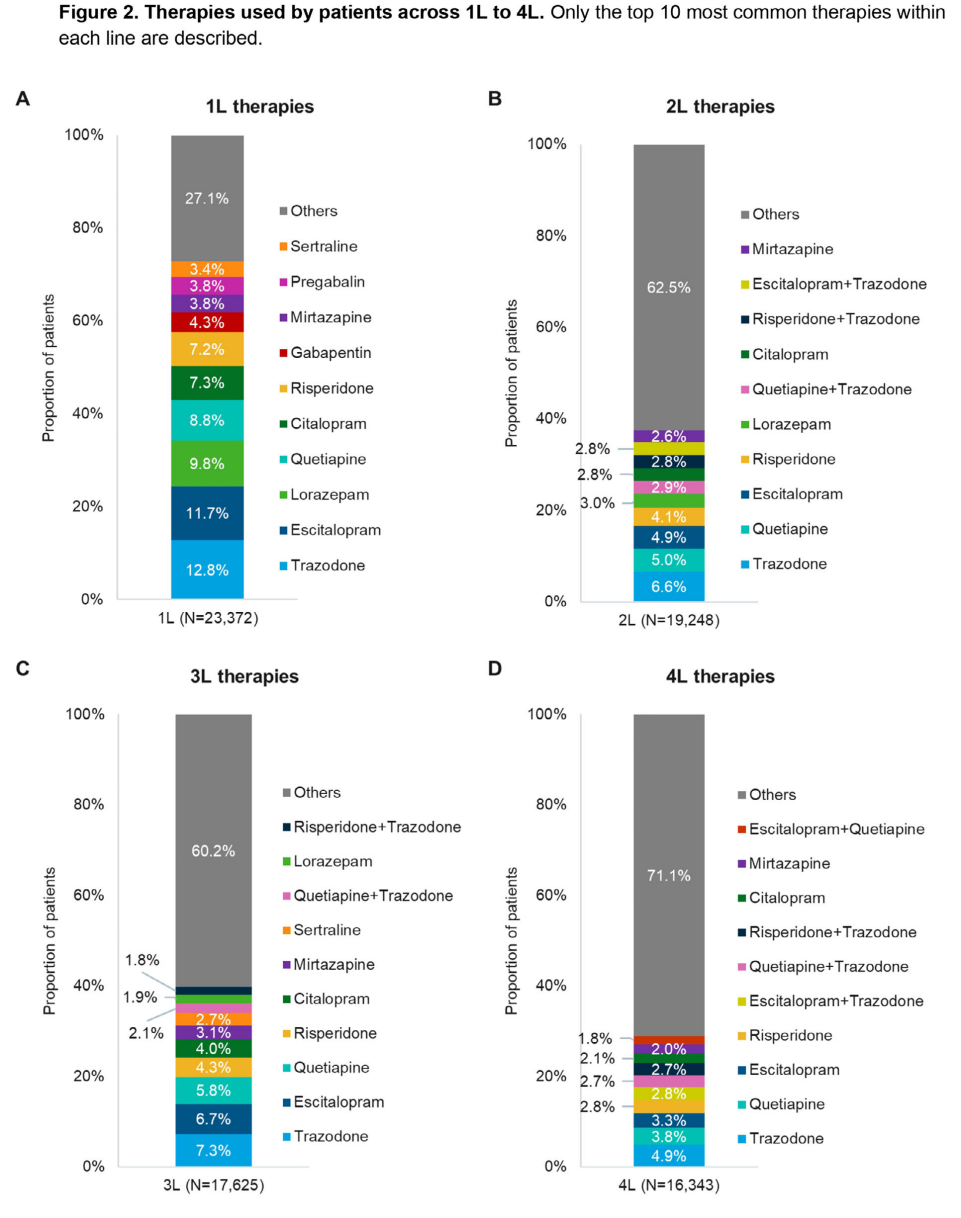# Treatment Patterns of Agitation associated with Alzheimer's Dementia (AAD) Patients in Canada

**DOI:** 10.1002/alz70858_098409

**Published:** 2025-12-24

**Authors:** Veronique Littmann, Francois Therrien, A. Marilise Marrache, Ceryl Tan, Natalie Nightingale, Calum S Neish, Zahinoor Ismail

**Affiliations:** ^1^ Otsuka Canada Pharmaceuticals Inc., Saint‐Laurent, QC, Canada; ^2^ Lundbeck Canada Inc., Saint‐Laurent, QC, Canada; ^3^ IQVIA Solutions Canada Inc., Mississauga, ON, Canada; ^4^ Hotchkiss Brain Institute, University of Calgary, Calgary, AB, Canada

## Abstract

**Background:**

Agitation associated with Alzheimer's dementia (AAD) is a challenging behavioral feature of Alzheimer's dementia (AD) characterized by excessive motor activity, verbal aggression, and physical aggression. Affecting up to 80% of people with AD, AAD is linked with greater caregiver burden, morbidity, and mortality. Due to a paucity of approved AAD treatments, therapeutic strategies may vary across clinicians. To gain insights into clinical practice, we examined the treatment patterns of AAD patients in Canada.

**Method:**

This was a retrospective cohort study using public drug plan claims data from Ontario and New Brunswick between 2003 and 2023 to characterize lines of therapy (LoTs). An indication algorithm was developed with a clinical expert to identify AAD through prescription claims for cognitive enhancers and medications commonly used to treat AAD symptoms (anticonvulsants [AC], antidepressants [ADT], antipsychotics [AP], benzodiazepines [benzo]). These inferred patients were indexed on the date of their first AC/ADT/AP/benzo and analyzed until their 4^th^ LoT.

**Result:**

Overall, 23,732 inferred AAD patients were identified, mostly from Ontario, with a median (IQR) age of 80 (11) years (Table 1). Overall, 70% of the cohort were followed until the end of their 4^th^ LoT (4L), and the proportion of those in a long‐term care setting increased from 14% (1L) to 54% (4L) (Figure 1). Inferred AAD patients were treated with a wide range of therapies, with over 500 to 2000 unique combinations observed across 1L to 4L, respectively. However, some medications and their combinations consistently ranked among the top ten most common therapies across LoT: citalopram, escitalopram, gabapentin, lorazepam, mirtazapine, pregabalin, quetiapine, risperidone, sertraline, and trazodone (Figure 2). The top ten therapies accounted for 73% of all 1L treatment approaches, with only risperidone indicated at that time for managing aggression and psychotic symptoms in severe AD. The highest proportion of patients remained on their 3L‐4L therapy after one year; the majority (≥80%) were adherent to these medications across all LoTs.

**Conclusion:**

There is considerable variability in AAD‐related treatment approaches among Canadian clinicians. Our findings highlight the need for more evidence on treatments specifically indicated for AAD, and education to facilitate evidence‐based clinical practice.